# Gastric cancer peritoneal metastasis: a bibliometric study from 2000 to 2024 using VOSviewer software

**DOI:** 10.3389/fonc.2025.1489043

**Published:** 2025-03-04

**Authors:** Manting Y, Dongfang L

**Affiliations:** ^1^ College of Integrative Medicine, Hunan University of Chinese Medicine, Changsha, China; ^2^ Hunan Cancer Hospital, Xiangya School of Medicine, Central South University, Changsha, Hunan, China

**Keywords:** gastric cancer, peritoneal metastasis, bibliometric analysis, VOSveiwer, knowledge-map

## Abstract

**Background:**

Gastric cancer remains a prevalent malignancy worldwide, with peritoneal metastasis being the predominant form of recurrence and metastasis, which are clear predictors of prognosis. The aim of this comprehensive bibliometric analysis was to assess the current status of the research landscape and to identify impending trends in gastric cancer peritoneal metastasis (GCPM).

**Methods:**

Relevant studies of GCPM were retrieved from the Web of Science Core Collection database. Qualified articles were screened based on the inclusion and exclusion criteria for further analysis. The selected publications were then subjected to bibliometric analysis utilizing VOSviewer software.

**Results:**

In total, 1,100 publications were included for analysis. The results revealed a consistent upward trend in the number of publications annually from 2000 to 2024, with an anticipated continuation of this growth in future research. The National Cancer Center Japan, emerged as the institution with the most publications and Professor Kodera and *Annals of Surgical Oncology* were identified as the most influential author and journal, respectively, in the domain of GCPM. In terms of international collaborations, the USA, Japan, and France were the most engaged countries. Yonemura was recognized as the most frequently cited author. Gastrectomy, systemic chemotherapy, and intraperitoneal therapy are the current research hotspots within this domain.

**Conclusion:**

Research related to GCPM had rapidly increased over the past two decades. These findings identify the most influential countries, institutions, authors, journals, and academic collaboration networks, while also clarifying hotspots and future trends in GCPM research.

## 1 Introduction

Gastric cancer (GC) is currently the fifth most common cancer and the third leading cause of cancer-related death worldwide, although the incidence and mortality rate are expected to continue to increase due to high-risk and aging populations (1, 2). Despite efforts to prolong survival and improve quality of life, current methods have limited efficacy against metastasis from advanced GC (3). Therefore, a comprehensive understanding of the underlying mechanisms, treatment strategies, and prognosis of GCPM is essential for effective management of advanced GC. The bibliometric technique was utilized in the present study to visually analyze GCPM studies included in the Web of Science Core Collection (WOSCC) database published between January 1, 2000 and March 18, 2024 to clarify progress in research, identify current hot topics and potential research areas of GCPM. At the same time, through visual analysis, the knowledge structure and trend of GCPM academic research are intuitively displayed, providing comprehensive reference and targeted guidance for subsequent researchers.

## 2 Research methods

This bibliometric study was conducted in accordance with the general guidelines proposed in a previous report (4). Once the purpose and scope of the study were established, relevant studies were retrieved from the WOSCC database and the target data were collected, analyzed, and reported.

### 2.1 Determining the purpose and scope of the study

The bibliometric evaluation criteria include two parts: “performance review” and “in the field of research” (5). Therefore, the aim of this study was to clarify trends in GCPM research in terms of authors, institutions, and countries. In terms of science, current trends in the treatment of GCPM were assessed to predict future hotspots. As another aspect of the first step, the scope of bibliometric research should be sufficiently large. Specifically, more than 500 publications in a certain field are expected.

### 2.2 Selecting bibliometric analysis techniques

The title, first author, year of publication, country, institution, keywords, and citations were selected for performance analysis. VOSviewer software 1.6.18 (https://www.vosviewer.com/) was employed to analyze the collected data for construction and visualization of bibliometric networks to reveal research trends and hotspots (6).

### 2.3 Collection of bibliometric data

The WOSCC database is a collection of more than 12,000 high-impact and high-quality scientific journals. In addition, the data retrieved from the WOSCC database are adaptable to most software for bibliometric analysis.

The keywords used to search the WOSCC database for articles related to peritoneal metastasis of GC included (“stomach” OR “gastric”) AND (“cancer” OR “tumor”) OR “oncology” OR “neoplasm” OR “carcinoma”) AND (“peritoneal metastasis” OR “peritoneal metastases”) AND (gastric cancer peritoneal metastasis). The date of publications was set from January 1, 2000 to March 16, 2024.

**Figure 1:**
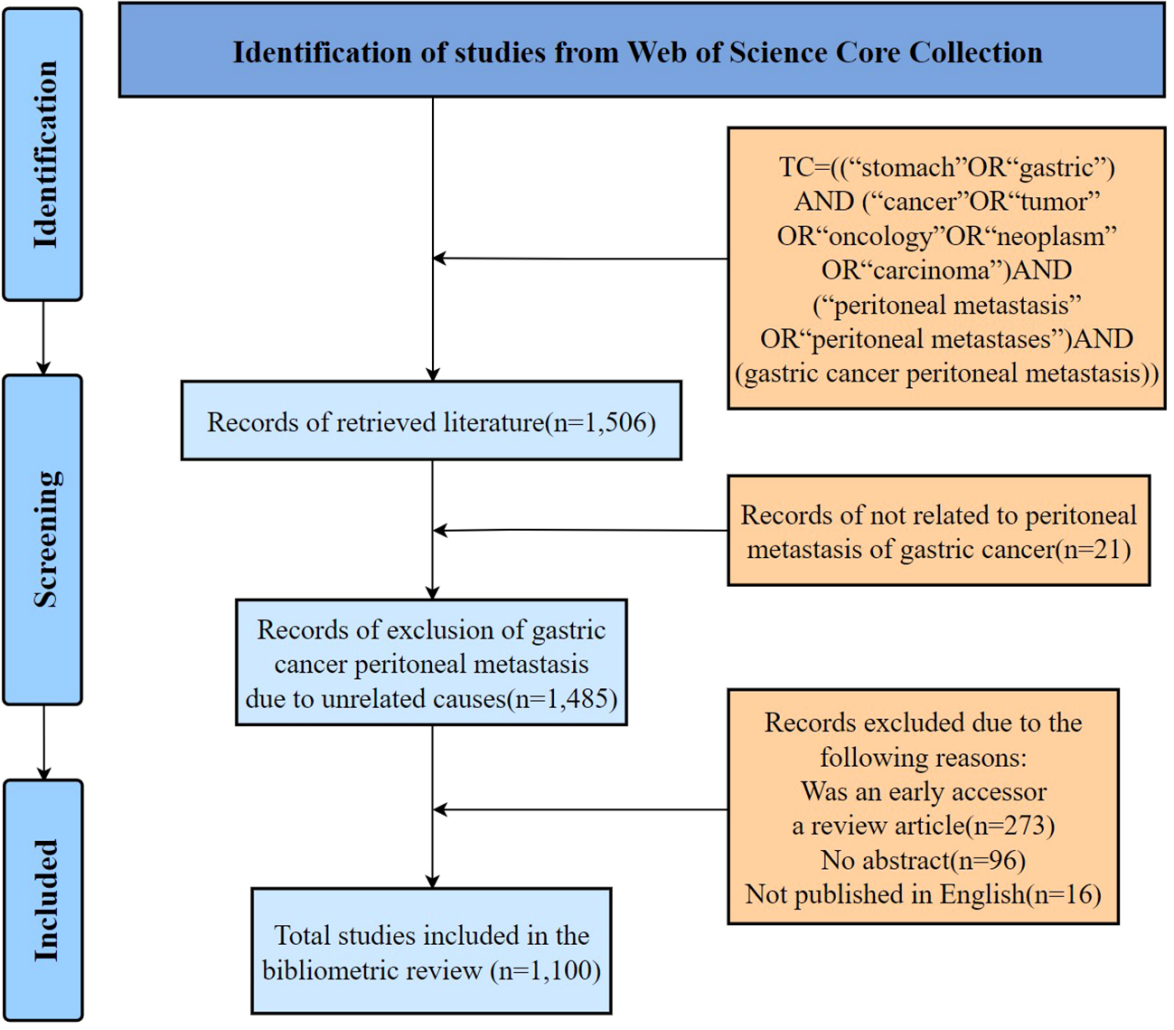
A flow chart of the data filtration process.

The screening and selection processes of this study were conducted in accordance with the Preferred Reporting Items for Systematic Reviews and Meta-Analyses (PRISMA) guidelines (**Figure 1**), which were established in 2009 to improve the quality of reporting of systematic evaluations and meta-analyses through the development of standardized reporting guidelines. The benefits of this approach are enhanced transparency and replicability of research, which enhance the overall credibility of scientific evidence (7). To ensure the relevance and quality of the included studies, literature screening and proofreading were independently conducted by two researchers. The inclusion criteria were articles (1) related to peritoneal metastasis of GC, (2) published after strict peer review, and (3) written in English, while the exclusion criteria were articles (1) not related to peritoneal metastasis of GC, (2) an “early access” or review, (3) lacking an abstract, and (4) not published in English. In order to avoid bias caused by daily updates to the database, all searches and data collection were performed on the same day. Finally, 1,100 articles met the inclusion criteria.

### 2.4 Running analysis and reporting research results

Keyword substitution was conducted to standardize the data and enhance the clarity and readability of the visualizations. Clear use of terminology allows for greater precision of the generated charts, network diagrams, and other visualizations to ensure the reliability of the results. Keywords have been replaced and optimised for this study.

## 3 Results and analysis

### 3.1 Analysis of annual distribution

**Figure 2:**
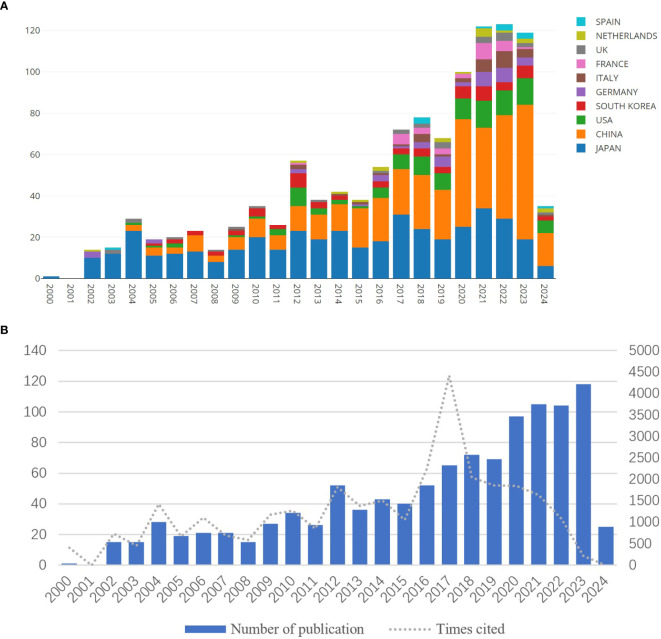
Number of publications per year **(A)** Annual distribution of articles **(B)** Publications per year by country.

**Figure 2** shows the number of publications on GCPM per year since 2000. Although incomplete, the number of publications was relatively lower in 2024. As indicated in **Figures 2A, B**, GCPM research has tended to increase annually, with the highest number of articles issued by groups in Japan, China, and the USA. Notably, the number of publications first exceeded 50 in 2012 and the publication volume has consistently been above 50 articles since 2016, demonstrating a clear increasing trend. Notably, the number of citations consistently exceeded 1,000 from 2012 to 2022, with a peak occurring in 2017.

Overall, as illustrated in **Figure 2**, research in this area went through two phases. During the first phase from 2000 to 2017, the publication and citation counts were relatively stable with limited growth, while the second phase began in 2018. In particular, 2017 saw a surge in citations, mainly due to the 89th Annual Meeting of the Japanese Gastric Cancer Association (JGCA) held in Hiroshima in 2017. In addition, the fourth edition of the *Japanese Guidelines for the Treatment of Gastric Cancer*, which was released in 2017, provides an authoritative standard for cross-sectional evaluation of the efficacy of GCPM treatment on a global scale. Afterward, the numbers of publications and citations have significantly increased.

### 3.2 Major countries and institutions contributing to GC research

The top 10 research organizations in terms of the number of related publications from 2000 to 2024 are listed in **Table 1**. Over the past 20 years, the 10 most influential institutions published 384 publications, accounting for 34.9% of the total. The country with the most organizations in terms of number of publications was Japan (n=237), followed by China (n=121). Among the institutions in Japan and China, National Cancer Center Japan and Shanghai Jiao Tong University published the most articles with 50 and 48, respectively.

**Table 1:** Top 10 research organizations in terms of number of publications.

Rank	Institution	Country	Number of publications
1	National Cancer Center Japan	Japan	50
2	Nagoya University	Japan	49
3	Shanghai Jiao Tong University	Peoples R China	48
4	Aichi Cancer Center	Japan	45
5	China Medical University	Peoples R China	45
6	University Of Tokyo	Japan	44
7	Sun Yat Sen University	Peoples R China	28
8	University Of Texas System	USA	26
9	Osaka Metropolitan University	Japan	25
10	Jichi Medical University	Japan	24

### 3.3 Top 10 journals in terms of number of publications

**Figure 3:**
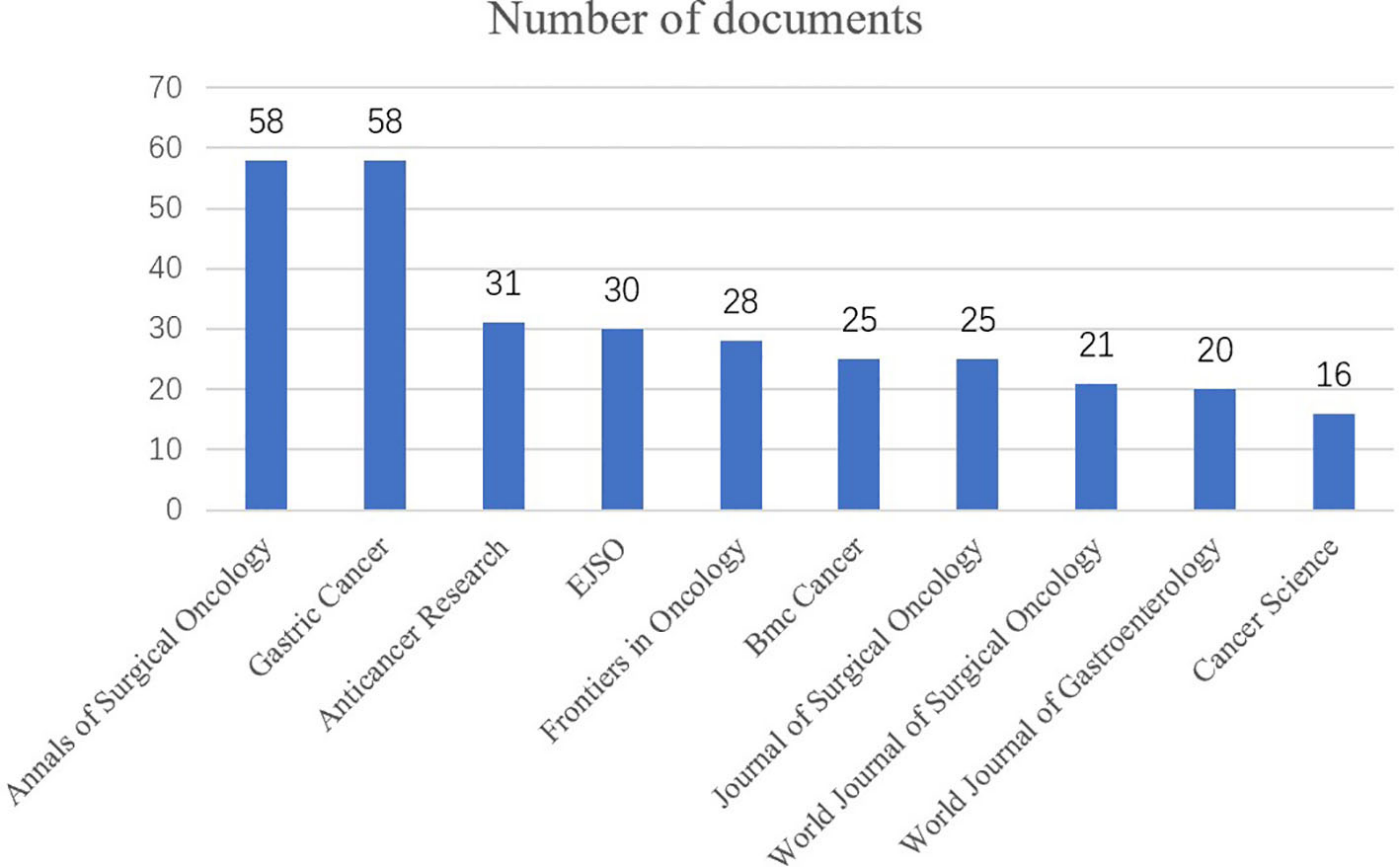
Top 10 journals with the highest number of publications.

The top 10 journals by publication count, encompassing 312 articles, which accounted for 28.1% of all related publications, are listed in **Figure 3**. The highest numbers of articles were published by *Annals of Surgical Oncology* and *Gastric Cancer*, accounting for 58 articles each. Also, each of the top 10 journals had published more than 15 articles on peritoneal metastases of GC.

### 3.4 Analysis of authors

The top 10 authors, comprising seven Japanese and three Chinese scholars, with the highest number of publications over the last 20 years are listed in **Table 2**. Collectively, these authors contributed 289 publications, which represent 26.3% of all related publications. Each of these authors published more than 20 articles. The top three authors were all from Japan, with Kodera having the most publications (n=47) and the highest *h*-index (68).

**Table 2:** Top 10 authors in terms of number of publications.

Rank	First Author	Organization	Number of publications	*h*-index
1	Kodera	Nagoya University	47	68
2	Kitayama	Jichi Medical University	42	43
3	Yamaguchi	Jichi Medical University	31	28
4	Zhang	Shanghai Jiao Tong University	26	13
5	Zhu	Shanghai Jiao Tong University	26	44
6	Kanda	Nagoya University	25	45
7	Ishigami	University of Tokyo	24	23
8	Li	Shanghai Jiao Tong University	23	13
9	Okamoto	Kyoto Prefectural University of Medicine	23	38
10	Tanaka	Nagoya University	22	31

### 3.5 Cooperation analysis

Collaboration analysis in bibliometrics is a method used to study the patterns and networks of cooperation among authors and countries as documented in publications to identify collaborative relationships by mapping co-authorships across articles (8). In this section, VOSviewer software was used to visualize and analyze the bibliometric data. The author and country were selected as units for cooperation analysis. The results of cooperation analysis provide insights into the hotspots and evolution of the field by highlighting cooperation among influential authors and countries (9).

#### 3.5.1 Author cooperation

The top 10 authors in terms of co-authorship are provided in **Table 3**. A quantitative overview of the influence of these authors in GC research is presented in **Table 4**. Kodera is the top-ranked author, with the highest total link strength of 187, 39 publications, and 1057 citations. These achievements indicate that this author is active and renowned in collaboration, thereby having a significant impact in the field. Kitayama is ranked second, with slightly fewer publications than Kodera (n=36), but a higher citation tally of 1191, indicating that Kitayama has garnered broader recognition and is frequently cited to bolster the studies of fellow scholars. Kanda is ranked third, with a total link strength of 144, 23 publications, and 510 citations. Kodera, who is ranked first in terms of number of publication, has strong collaborative relationships with Kitayama, Kanda, and Yamaguchi. Also, there were two robust academic clusters, spearheaded by Kodera, Kitayama, Kanda, and Yamaguchi. Clarification of the cooperation among different authors can help to identify potential opportunities for collaboration, thus promoting the integration of knowledge and innovation in the field of GCPM.

**Table 3:** Top 10 most prolific cooperating authors in terms of numbers of publications and citations.

Rank	Author	Total link strength	Publications	Citations
1	Kodera	187	39	1057
2	Kitayama	149	36	1191
3	Kanda	144	23	510
4	Yamaguchi	141	29	870
5	Tanaka	126	18	423
6	Hayashi	104	14	348
7	Kobayashi	95	13	375
8	Yamada	87	12	450
9	Ishigami	86	22	878
10	Fujiwara	81	12	359

**Table 4:** Top 10 countries in terms of numbers of publications and citations.

Rank	Country	Total link strength	Publications	Citations
1	USA	105	107	2626
2	Japan	75	422	13956
3	France	66	28	1108
4	Germany	58	40	1333
5	England	56	26	1251
6	Peoples R China	48	405	6912
7	Italy	41	33	654
8	Belgium	30	6	407
9	South Korea	30	63	1926
10	Australia	27	8	293

#### 3.5.2 Country cooperation

The cooperative relationships among countries are shown in **Table 4**. The USA had the highest total link strength (105), demonstrating significant leadership in GCPM research. Japan had the highest number of publications and citations, indicating active research activities and high academic impacts. Notwithstanding, France produced fewer publications than the USA and Japan, although the total link strength and citation count indicate high-quality research. Notably, the high numbers of publication and citations reflect the expansive scope of research endeavors in China and robust engagement in international collaborations. These findings not only highlight the role and impact of each country within the global research community, but also facilitate evaluation and comprehension of the quality and effectiveness of the respective research networks.

### 3.6 Co-citation analysis

In the realm of bibliometric analysis, co-citation visualization is used to map out the intellectual structure of a particular research field, identify clusters of related publications, and reveal patterns of scholarly communication (10). By examining frequently co-cited articles, researchers can infer the degree of subject similarity and the network of knowledge within a specific discipline (11). This study employed co-citation analysis to reveal research trends, topic clustering, knowledge structures, and interdisciplinary intersections within the field of GCPM, which not only helps to interpret the relationships among academic endeavors, but also reveals the academic structure to assess academic impact (12).

#### 3.6.1 Journal co-citations

**Figure 4:**
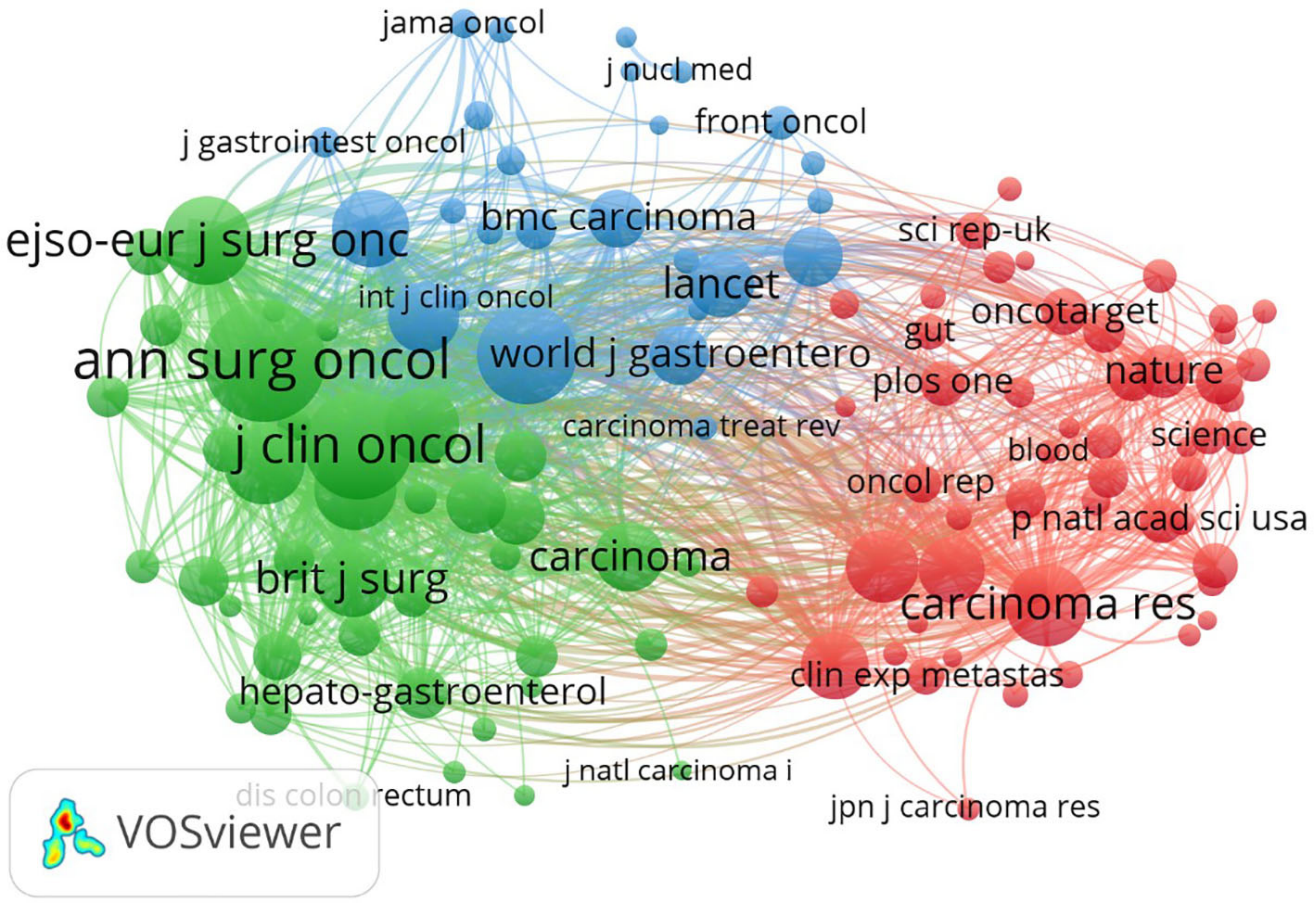
Journal co-citations network.

**Figure 5:**
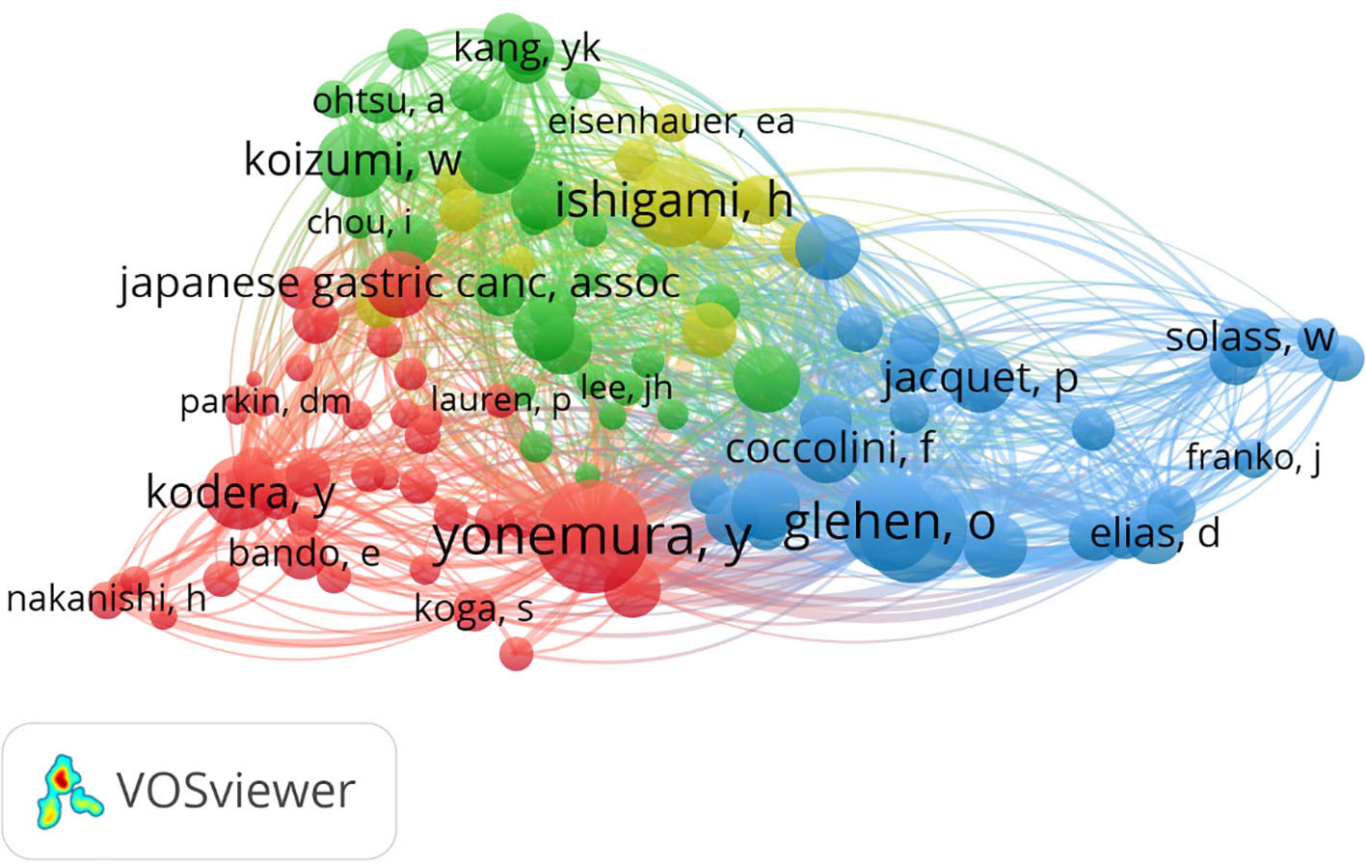
Reference co-citation networks.

A co-citation map was generated with VOSviewer software. The type of analysis and designated journal were selected as units for co-citation analysis. The minimum number of documents was set at 50. The results are presented in **Table 5**, **Figure 4**. As shown in **Figure 5**, journals that published articles related to peritoneal metastasis of GC were closely linked, forming three clusters based on co-citations. The three clusters are very closely linked to each other. The top 10 co-cited journals with the highest total link strength are listed in **Table 5**. *Annals of Surgical Oncology* and *Journal of Clinical Oncology* have the highest total link strength, suggesting huge influences on GCPM research. *Gastric Cancer*, despite having a lower total link strength than the previous two journals, has a considerably higher citation count, implying that the published articles likely have high levels of specialization and interest. The articles published by these journals are extensively accessed and applied, albeit with fewer specific references. This discrepancy might stem from the expansive nature of the research domains or the dispersed influence of the publications. *Annals of Surgery*, *The Lancet*. *Oncology*, *British Journal of Surgery*, and *New England Journal of Medicine* may not boast the highest total link strength or citation counts in GCPM research, but are generally held in high esteem within the field, suggesting that the published articles likely exert considerable influence across the broader medical landscape.

**Table 5:** Top 10 journal in terms of total link strength and number of citations.

Rank	Journal	Total link strength	Citations
1	*Annals of Surgical Oncology*	48043	1457
2	*Journal of Clinical Oncology*	37467	1260
3	*Gastric Cancer*	31667	1352
4	*EJSO*	25493	609
5	*Carcinoma Research*	21961	863
6	*Journal of Surgical Oncology*	21753	758
7	*Annals of Surgery*	20766	667
8	*The Lancet Oncology*	19066	616
9	*British Journal of Surgery*	17856	662
10	*New England Journal of Medicine*	17721	502

#### 3.6.2 Top authors in terms of total link strength and number of co-citations

**Figure 6:**
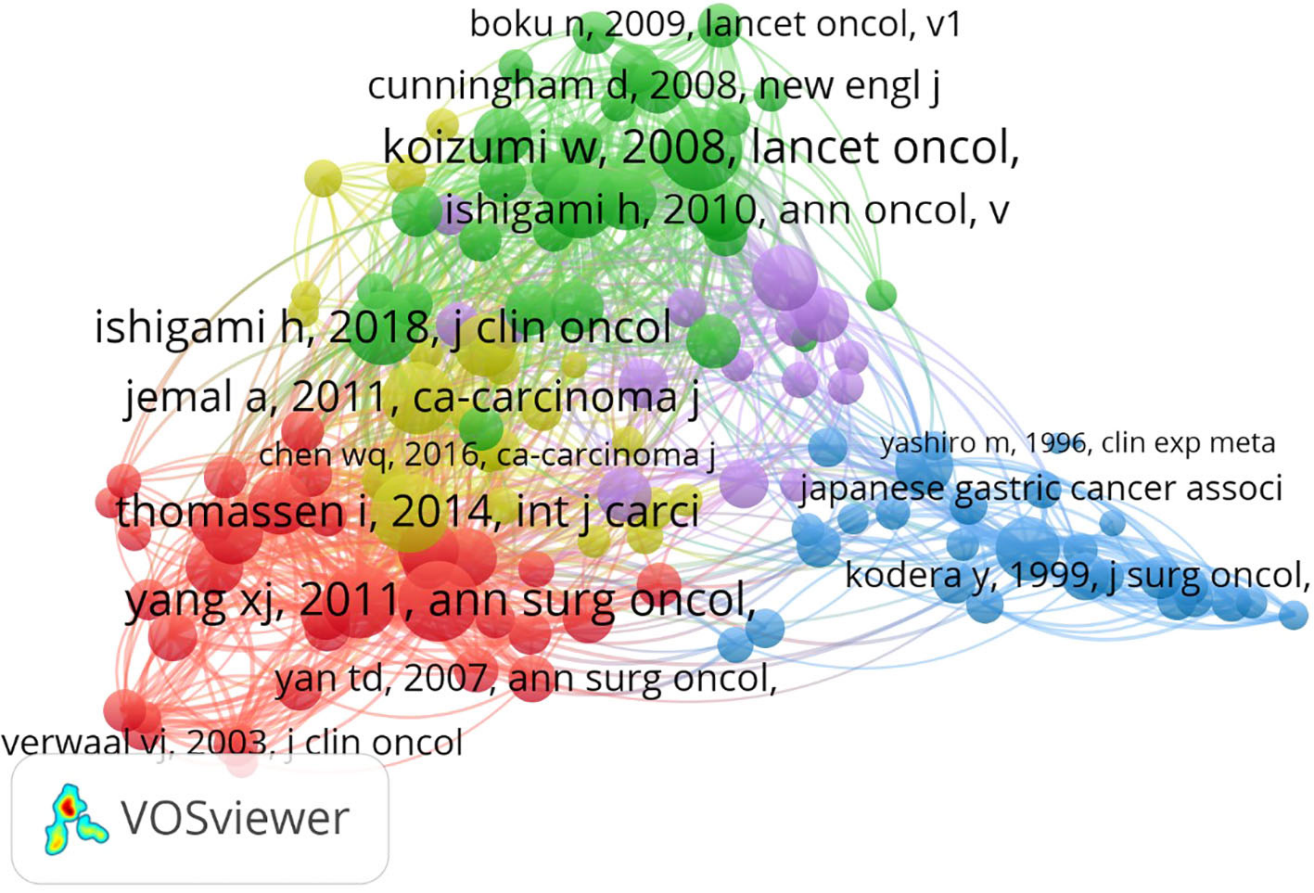
Author co-citations network.

Co-citation analysis was conducted to reveal clusters or networks of authors in the field (13). As shown by the results presented in **Figure 6**, the author co-citation network contained four closely interconnected clusters, highlighting the associations between principal researchers within the field of GC research. Specifically, the first cluster included the authors Yonemura and Kodera, whose research significantly intersects, demonstrating common interests and deep collaboration on related topics. The second cluster is a tight collaborative relationship between Bang and Koizumi, indicating complementary research. The third cluster, centered around Ishigami, not only encompasses the main contributions of this author, but also exhibits good connectivity with other researchers, reflecting a pivotal role within the academic network. Finally, the fourth cluster consists of Sugarbaker, Yang, and Coccolini, whose collaborative efforts across various geographical regions underscore active roles in international research collaborations. Overall, these clusters clarify the collaboration and citation patterns among key researchers in the field, providing a valuable perspective to understand the structure of the academic network.

As shown in **Table 6**, Japan has the most representative researchers (n=5) based on total link strength. The top three researchers were Yonemura (*h*-index=40), Sugarbaker (*h*-index=74) and Glehen (*h*-index=56). Yonemura was ranked first in total link strength (4,088) and citations (341), underscoring a pivotal role in GC research and widespread recognition within the academic community. The link strength of the second-ranked American scholar, Sugarbaker, was slightly lower than that of the top-ranked scholar, indicating equally relevant and influential research. Authors in the GCPM field have formed a large network composed of strong connections, with good connectivity and no apparent isolated points, demonstrating that each scholar has been co-cited with others and good commonality in research among the authors.

**Table 6:** Top 10 authors in terms of total link strength and number of co-citations.

Rank	Author	Country	Total link strength	Citations
1	Yonemura	Japan	4088	341
2	Sugarbaker	USA	3641	284
3	Glehen	France	2929	186
4	Ishigami	Japan	2505	216
5	Kodera	Japan	1741	200
6	Koizumi	Japan	1737	158
7	Bang	South Korea	1555	141
8	Yang	Peoples R China	1487	94
9	Japanese Gastric Cancer Association	Japan	1441	149
10	Coccolini	Italy	1433	99

#### 3.6.3 Reference co-citations

As demonstrated in **Figure 5**, **Table 7**, the results of reference co-citation analysis revealed strong connections among references, although the main focus was limited to three key research subdomains: treatment, epidemiology, and guidelines for GC.

**Table 7:** Top 10 reference co-citations in terms of total link strength and number of citations.

Rank	Cited references	Author	Total link strength	Citations	Year
1	S-1 plus cisplatin versus S-1 alone for first-line treatment of advanced gastric cancer (SPIRITS trial): a phase III trial.	Koizumi	861	110	2008
2	Cytoreductive surgery and hyperthermic intraperitoneal chemotherapy improves survival of patients with peritoneal carcinomatosis from gastric cancer: final results of a phase III randomized clinical trial.	Yang	785	79	2011
3	Peritoneal carcinomatosis of gastric origin: a population-based study on incidence, survival and risk factors.	Thomassen	773	119	2014
4	Phase III Trial Comparing Intraperitoneal and Intravenous Paclitaxel Plus S-1 Versus Cisplatin Plus S-1 in Patients with Gastric Cancer with Peritoneal Metastasis: PHOENIX-GC Trial.	Ishigami	683	78	2018
5	Trastuzumab in combination with chemotherapy versus chemotherapy alone for treatment of HER2-positive advanced gastric or gastro-oesophageal junction cancer (ToGA): a phase 3, open-label, randomised controlled trial.	Bang	682	91	2010
6	Peritoneal carcinomatosis from gastric cancer: a multi-institutional study of 159 patients treated by cytoreductive surgery combined with perioperative intraperitoneal chemotherapy.	Glehen	673	69	2010
7	Global Cancer Statistics	Jemal	638	138	2011
8	Japanese classification of gastric carcinoma: 3rd English edition	Japanese Gastric Cancer Association	520	75	2011
9	Intraoperative lavage for cytological examination in 1,297 patients with gastric carcinoma.	Bando	510	77	1999
10	Gastrectomy plus chemotherapy versus chemotherapy alone for advanced gastric cancer with a single non-curable factor (REGATTA): a phase 3, randomised controlled trial.	Fujitani	490	61	2016

Within the subdomain of medical treatment, the effects of S-1 plus cisplatin versus S-1 alone for treatment of advanced GC were compared (14), highlighting the importance and wide recognition in GC treatment research (total link strength=861, citations=110). In addition, studies by Yang, Ishigami, Bang, Glehen, Bando, and Fujitani also warranted intense attention.

Within the subdomain of epidemiology, population-based data on peritoneal cancers of gastric origin, which included the incidence, survival rates, and risk factors (15). Because peritoneal metastasis is a common complication of GC, it is important to clarify the epidemiology of GC. On the other hand, a prior report (16) that provided global statistics on cancer had the highest number of citations (138) despite a relatively low total link strength (638), indicating wide applicability and importance.

The “Gastric Cancer Classification Guidelines” published by the Japanese Gastric Cancer Association (17) is worthy of scholarly attention as indicated by the total link strength of 520 and 75 citations. These guidelines are widely adopted for classification of GC for research and diagnosis.

In conclusion, these studies cover various aspects of GC treatment, epidemiology, and diagnosis, providing valuable information and guidance for GC treatment and research with high reference value for scholars who are just beginning studies of peritoneal metastasis of GC.

### 3.7 Keyword co-occurrence analysis

**Figure 7:**
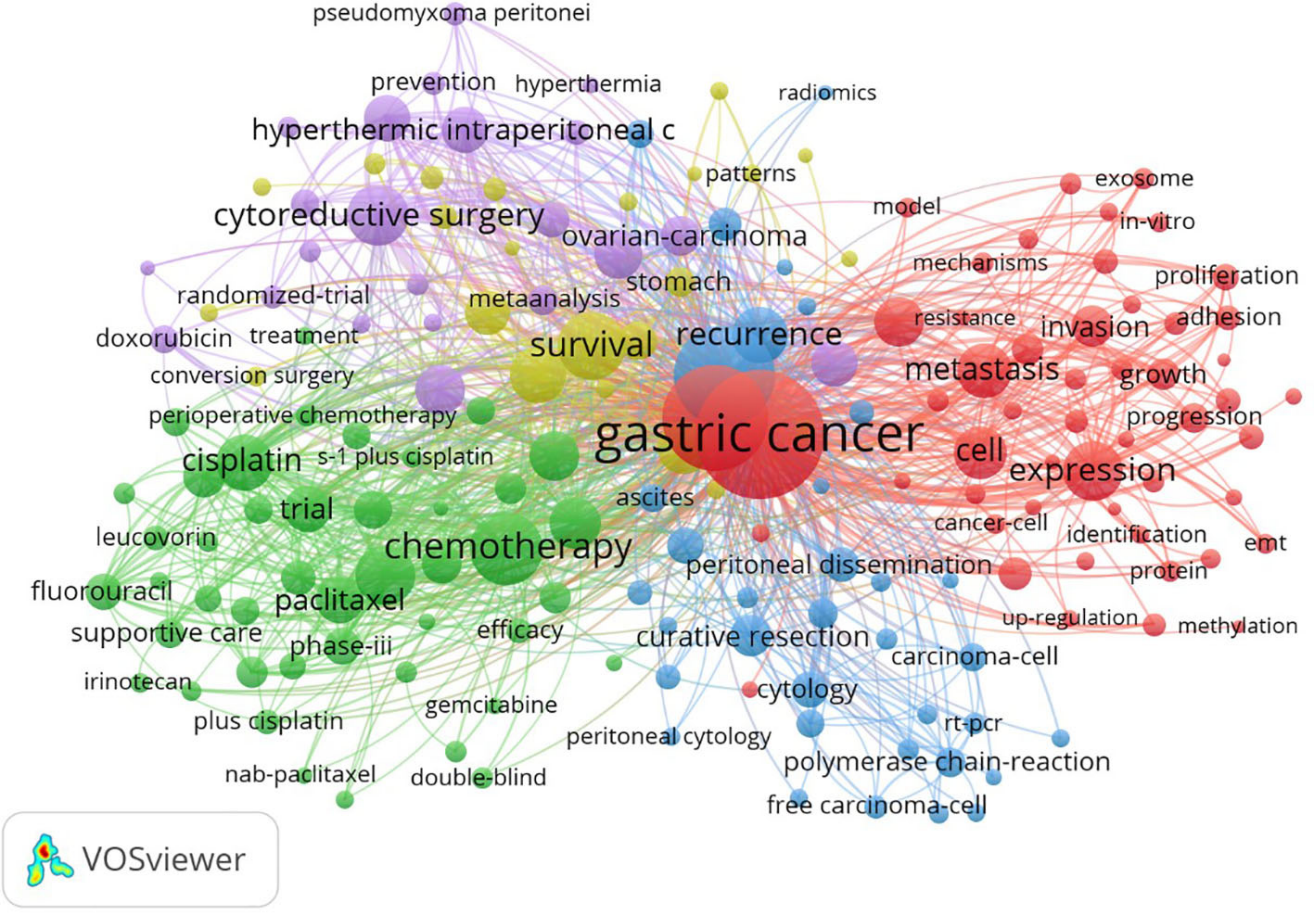
Keyword clustering network.

**Figure 8:**
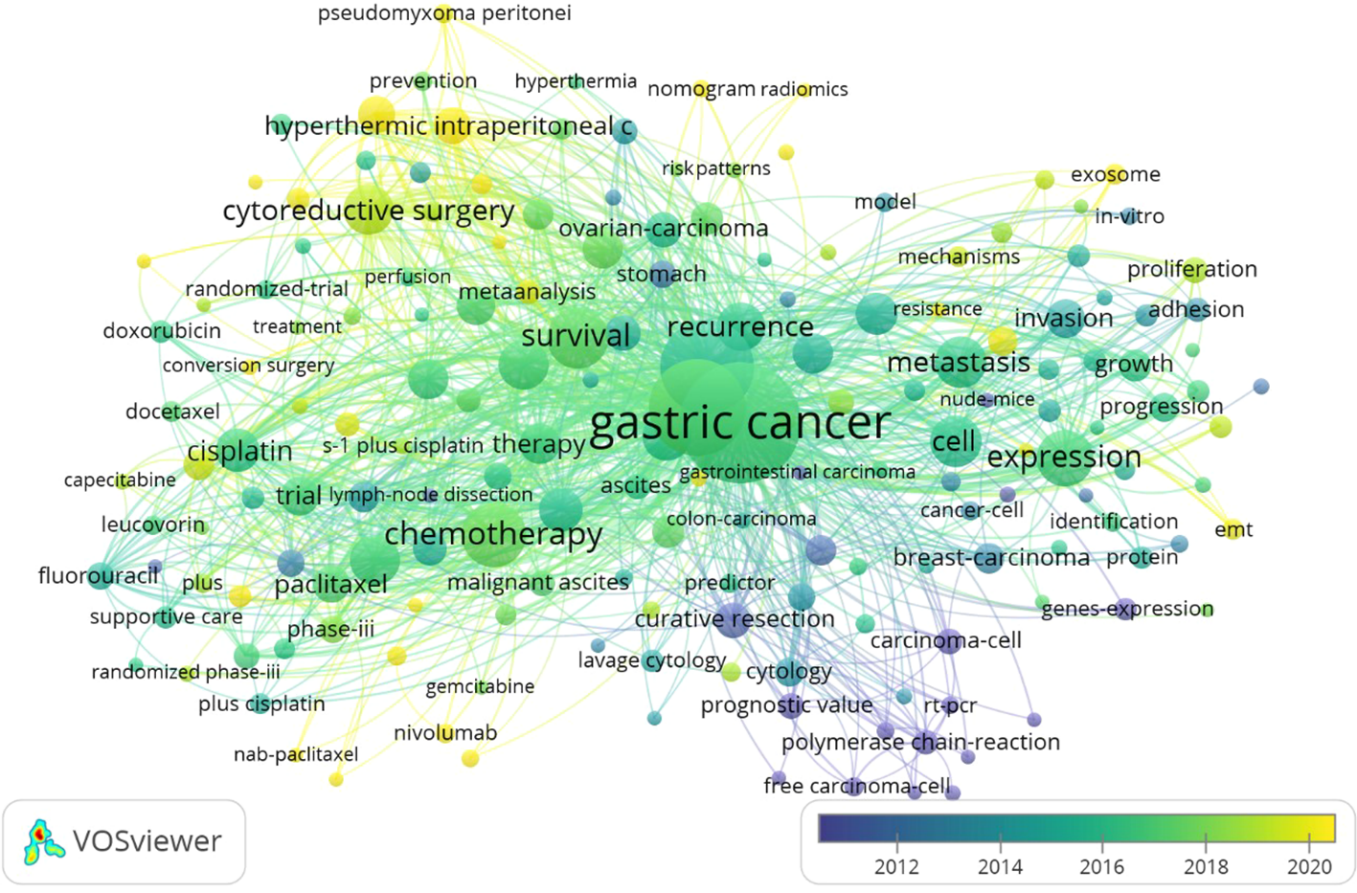
Keyword evolution network.

In total, 1,100 articles were analyzed with VOSviewer software. The terms “co-occurrence” and “keywords” were selected for analysis with “full counting” as the calculation method. As a result, 176 keywords that appeared more than 10 times were classified into five clusters (**Figure 7**). Keywords clustered by co-occurrence can reflect the main research directions and research hotspots in academia (4). As shown in **Figure 8**, cluster 1 (red) mainly centers around the molecular mechanisms related to GCPM, with keywords including *genes-expression*, *mechanisms*, and *proliferation*; cluster 2 (green) primarily details the subdomain of chemotherapy, including *cisplatin*, *paclitaxel*, and *gemcitabine*; cluster 3 (blue) discusses prognosis, including *recurrence*, *prognostic value*, and *diagnosis*; cluster 4 (yellow) focuses on the subdomain of gastric resection, comprising *survival*, *gastrectomy*, *surgery*, and *complications*; and cluster 5 (purple) addresses intraperitoneal treatment, including the keywords *hyperthermic intraperitoneal chemotherapy*, *perfusion*, and *cytoreductive surgery*.

#### 3.7.1 Red cluster: molecular mechanisms of GCPM

The red cluster, containing 51 keywords, focused on elucidating the molecular transfer mechanism of GCPM. High-frequency keywords included “metastasis,” “invasion,” “migration,” “dissemination,” “adhesion,” and “pathway.” This cluster primarily delves into the biological processes and molecular mechanisms of cancer cell dissemination from the primary tumor site to other parts of the body. The molecular transfer mechanism of GCPM stands as a research focal point in the GCPM domain, with scholars dedicated to unraveling the mechanisms related to disease onset. Research findings are poised to provide a scientific foundation for proposing novel treatment modalities for future clinical practice. Notably, previous research identified a correlation between enhanced expression of long non-coding RNAs (lncRNAs) and the occurrence of GC (18). As early as 1997, lncRNA-ATB was shown to enhance tumor cell adhesion to the peritoneum. More recent studies (19, 20) further established a significant association of lncRNAs with the invasion, migration, proliferation, and cycle of GC cells, ultimately leading to an unfavorable prognosis.

Additionally, some low-frequency keywords warrant attention, such as “biomarker,” “marker,” “gene expression,” “microRNA,” and “fibroblasts.” These keywords, elucidating the biomarkers and molecular therapeutic targets of GCPM, are intended to identify specific molecules as potential diagnostic or prognostic targets. For example, a microRNA was identified as a biomarker for peritoneal recurrence after curative surgery for GC, thus offering a new method for early diagnosis of GCPM (21, 22). Additionally, metabolites related to exosomes and cancer-associated fibroblasts are also considered ideal biomarkers for early diagnosis of GC (23, 24). Since these keywords are less frequently discussed, but with high co-occurrence, this topic, although under-researched, likely has significant potential in GCPM research. Understanding the regulation of various molecules in cancer present potential targets to halt disease progression, while providing valuable insights into the diagnosis and treatment of therapy-resistant GCPM.

#### 3.7.2 Green cluster: chemotherapy

Chemotherapy and combination therapies for GC are the focal point of the green cluster. In total 44 high-frequency keywords were identified, which included “S-1,” “first-line treatment,” “adjuvant chemotherapy,” “neoadjuvant chemotherapy,” “chemotherapy,” “capecitabine,” “cisplatin,” “docetaxel,” “fluorouracil,” “gemcitabine,” “irinotecan,” “leucovorin,” “methotrexate,” “oxaliplatin,” “paclitaxel,” and “nab-paclitaxel.” These keywords highlight multifaceted chemotherapeutic agents and drug combinations used for initial and subsequent treatments, and also integrate preoperative (neoadjuvant) and postoperative (adjuvant) chemotherapies, in addition to assessment of the effectiveness for treatment of advanced GC, including strategies to improve survival rates and reduce tumor burden. Chemotherapy is a primary treatment modality for advanced GC (25). In order to improve clinical outcomes, researchers have been working to optimize treatment strategies over the last two decades through innovative chemotherapy regimens and rigorous clinical trials. Hence, this topic is expected to be a continued focal point of ongoing research. Over the past 20 years, researchers have conducted a series of clinical trials in search of more effective chemotherapies (26, 27). These trials aimed to evaluate the efficacy and safety of different chemotherapeutic drugs for treatment of GCPM. Specifically, scientists have sought to discover drug combinations to maximize survival rates, while minimizing side effects (14), covering not only traditional chemotherapeutic drugs, but also new drugs and therapies targeting GC cells. Personalization of treatment strategies is also an important goal of trials to enhance treatment effectiveness and quality of life.

#### 3.7.3 Blue cluster: diagnostic

The blue cluster focused on diagnostics. The high-frequency keywords in this cluster, which mainly related to the diagnosis of GCPM, included “ascites,” “carcinoembryonic antigen,” “computed tomography,” “cytology,” “diagnosis,” “lavage cytology,” “laparoscopy,” “peritoneal cytology,” “radiomics,” “staging laparoscopy,” “quantitative detection,” “polymerase chain reaction,” and “RT-PCR.” These keywords describe various diagnostic methods used to detect and assess the spread of GC to the peritoneum, involving imaging techniques (e.g., computed tomography), biomarkers (e.g., carcinoembryonic antigen), and procedures (e.g., laparoscopy and peritoneal lavage cytology). These diagnostic tools can assist confirmation of the presence of free cancer cells and assess the extent of disease to effectively stage the cancer. Accurate diagnosis of GCPM is a critically important and challenging component of GC management. Researchers employ a variety of methods to enhance the accuracy and timeliness of disease diagnosis. For instance, peritoneal lavage cytology is performed to assess the prognosis of GCPM patients as well as appropriateness for resection (28). Detection of specific markers in ascites is employed to judge the clinical prognosis of advanced GC (29, 30). Overall, while there has been progress in successful diagnosis of peritoneal metastasis of GC, an array of challenges still exists. Researchers are working to improve the sensitivity and specificity of diagnosis to accurately identify and manage this complex disease at an early clinical stage.

#### 3.7.4 Yellow cluster: surgical treatment

The yellow cluster primarily discusses surgical treatment of GCPM, with 23 high-frequency keywords, such as “gastrectomy,” “surgery,” “surgical treatment,” “resection,” “conversion surgery,” and “complications.” The keywords identified various surgical methods for treatment of GC as the focal research topic, including the removal of all or part of the stomach (gastrectomy) and other innovative surgical techniques designed to convert inoperable to operable cases. Radical gastrectomy a common procedure for advanced GC, although efficacy for GCPM remains controversial. Gastrectomy can improve survival, decrease clinical symptoms, and enhance quality of life of GCPM patients (31, 32). Notably, the effects of gastrectomy on treatment outcomes for GCPM were not recognized by several researchers (33, 34). Therefore, the value of gastrectomy for GCPM remains controversial within the medical community. Although gastrectomy offers potential survival benefits in some cases, there is no broad consensus on the applicability and effectiveness for GCPM. The controversy over this treatment method may drive future research to assess the actual benefits of gastrectomy and identify patient groups most likely to benefit. By providing clinicians with a solid base of evidence, future research could help select appropriate strategies for treatment of GCPM, thereby gradually resolving the controversy and challenges of gastrectomy for GCPM to improve management of this complex clinical disease.

#### 3.7.5 Purple cluster: intraperitoneal treatment

The purple cluster primarily describes intra-abdominal therapies, with high-frequency keywords including “cell regeneration surgery,” “hyperthermic intraperitoneal chemotherapy (HIPEC),” “intraperitoneal chemotherapy,” “docetaxel,” “thermal therapy,” “low-dose cisplatin,” and “mitomycin C,” among others. These keywords focus on specialized treatment regimens that combine surgery with intraperitoneal hyperthermic chemotherapy to directly target the abdominal cavity. This approach is particularly suitable for treatment of peritoneal metastasis of GC. Due to the presence of the blood-peritoneal barrier, the efficacy of systemic chemotherapy for treatment of GCPM is not satisfactory (35). Over the past two decades, researchers have been actively seeking better treatment strategies, which has led to the proposal of intraperitoneal therapy. The current forms of intra-abdominal therapy are diverse, with intraperitoneal chemotherapy, CRS, and HIPEC, being the most widely used (36). However, CRS and HIPEC for treatment of peritoneal metastasis of GC remains controversial. The results of a multicenter retrospective study showed that as compared to CRS alone, CRS with HIPEC significantly improved median overall survival and recurrence-free survival (37). Interestingly, another study reported that CRS with HIPEC was associated with an increased risk of isolated extraperitoneal recurrence, suggesting that HIPEC may be beneficial for specific patients (38). Overall, CRS with HIPEC can provide better clinical efficacy for GCPM patients who meet specific criteria. The keywords of this cluster suggest future research hotspots in the field of GCPM research. Specifically, with the continued improvement in drug delivery technology targeting the intraperitoneal cavity, such as the development of novel delivery systems and delivery routes, it will be possible to deliver therapeutic drugs more precisely to tumor sites, thereby improving efficacy. In addition, intraperitoneal therapy includes not only more targeted drug therapy but also the potential application of emerging treatment modalities, such as immunotherapy and targeted therapy.

**Figure 9:**
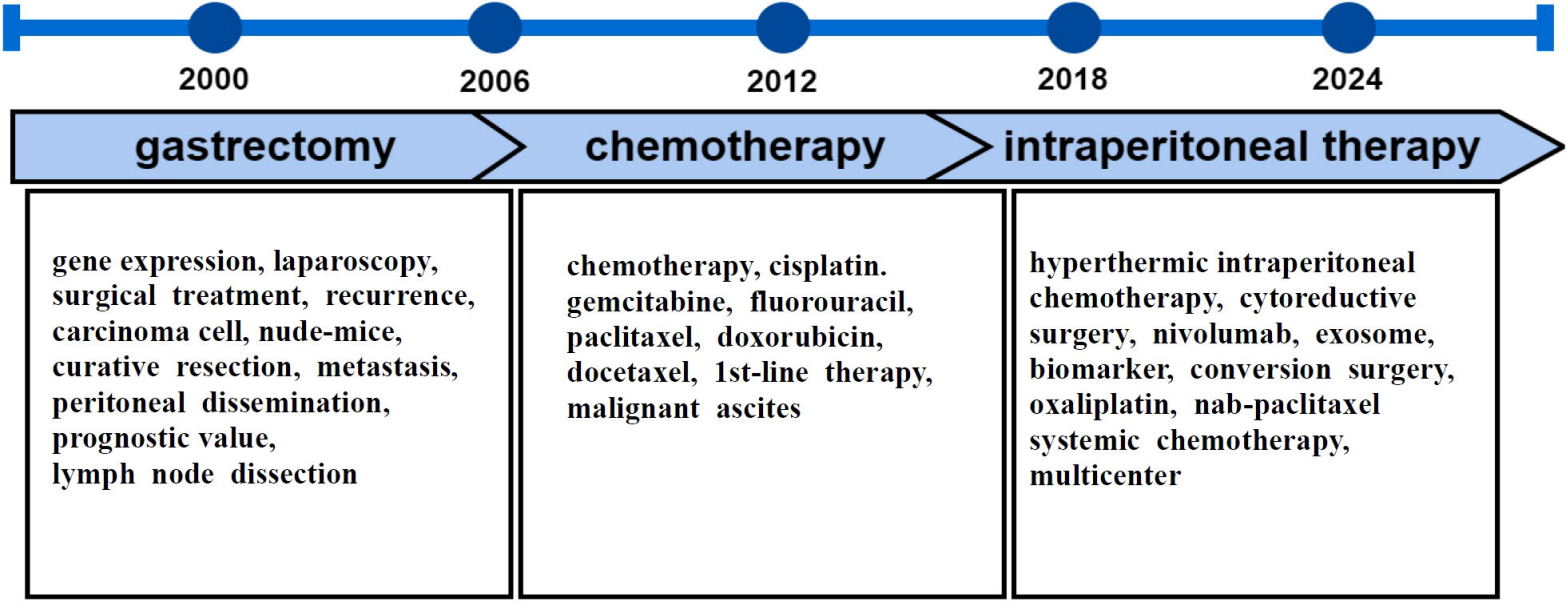
Clinical management of GCPM flowchart.

Overall, five categories relevant to GCPM research were unveiled based on the visualized results. It is evident, as implied by the dense connections between the different clusters in **Figure 8**, that GCPM treatments should not be viewed independently, but rather require fundamental and clinical research. The findings of these analyses provide important information for researchers to focus on current research trends and extend studies to broader categories in order to obtain a panoramic and in-depth view of this research domain. Based on the above results, we constructed a flow chart to optimize the clinical management of patients with gastric cancer peritoneal metastasis. In the flow chart, the patients were divided into three different categories according to the examination results, and the corresponding treatment plan was recommended for each category of patients (**Figure 9**).

### 3.8 Research evolution

**Figure 10:**
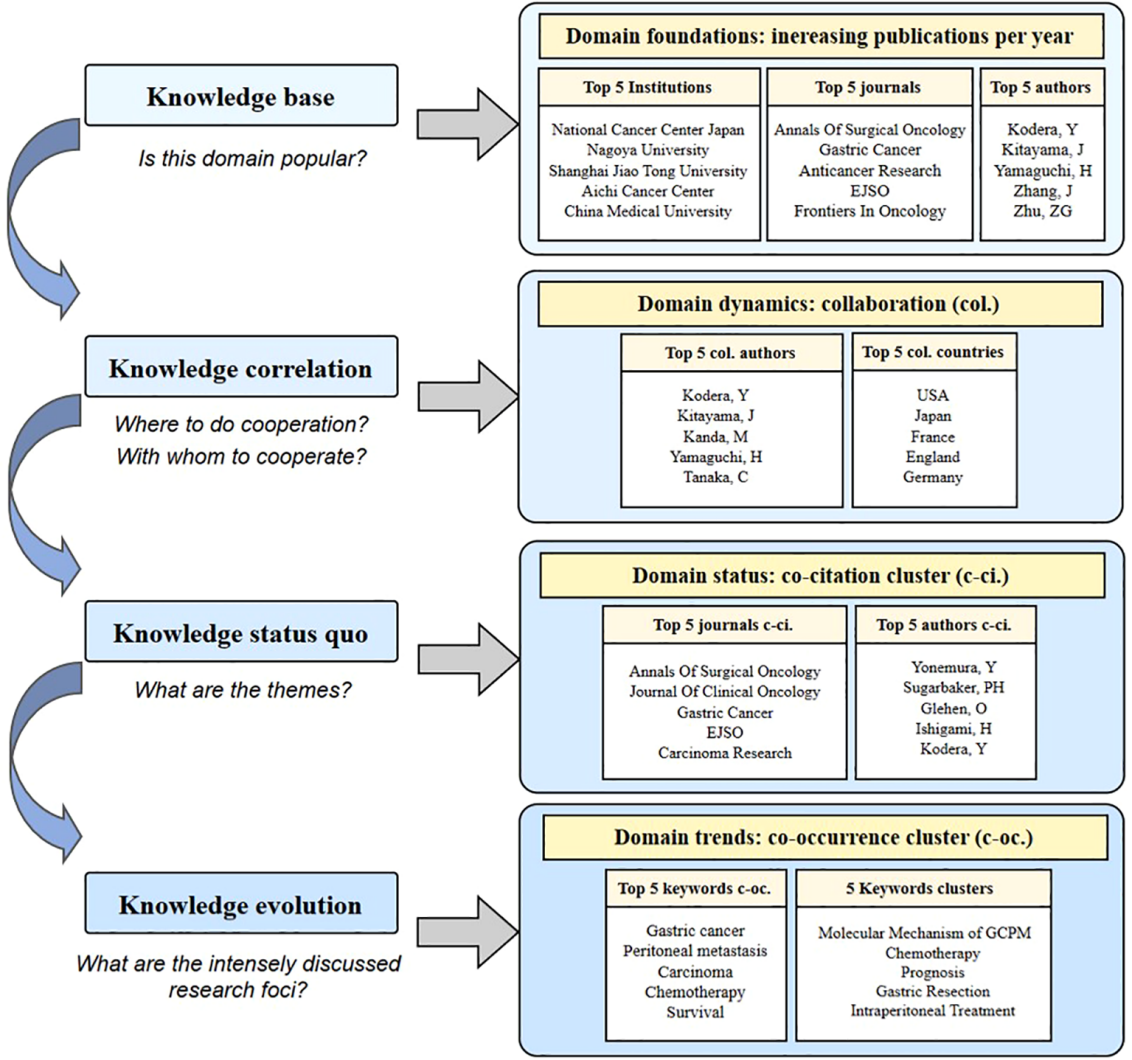
Research evolutionary routes.

The generalized keywords overlay map reveals the evolution of topics in GCPM research (**Figure 8**). Research topics between 2000 and 2024 emphasize three subdomains that occurred in chronological order: gastrectomy, chemotherapy, and intraperitoneal therapy (**Figure 10**).

During the predevelopment period (2001–2011), researchers were mainly concerned with the staging of GC and the progression of D2 surgery. At this phase, researchers continue to investigate mechanism underlying the recurrence and metastasis of GC (39). Combinations of chemotherapy and gastrectomy for treatment of advanced GC have shown that the median survival period after gastrectomy for advanced GC was superior to that of the non-surgical treatment group, with one- and two-year survival rates significantly higher than the non-surgical treatment group (35–40). Conversely, a phase 3 randomized controlled trial revealed that the addition of gastrectomy to chemotherapy may not confer significant clinical benefit over chemotherapy alone for patients with advanced GC presenting with a single incurable factor (34). Therefore, further studies are needed to determine whether radical gastrectomy has clinical value for patients with advanced GC or distant metastases.

Although surgery and other related treatments can improve the clinical prognosis of patients with early GC, the efficacy and prognostic outcomes of therapeutic measures for GCPM remain poor. Hence, research entered the second phase (2011–2019). In this stage, GC treatment entered the era of chemotherapy, targeted therapy, and immunotherapy. For patients with advanced GC, the National Comprehensive Cancer Network guidelines recommend systemic chemotherapy or optimal supportive care as the primary treatment option (38). The first-line chemotherapy regimen for stage IV GC for patients eligible for chemotherapy primarily involves the combination of cisplatin and fluoropyrimidine, with the addition of trastuzumab for patients who are positive for human epidermal growth factor receptor 2 (41). In addition, adjuvant chemotherapy was found to significantly improve disease-free survival after gastrectomy (42).

However, the GC treatment model based on radical gastrectomy combined with systemic chemotherapy may be flawed for GCPM. The primary tumor and surrounding lymph nodes are controlled by extended resection through radical GC surgery. Blood flow and lymph node metastases are influenced by systemic chemotherapy. However, research into regional implantation metastasis has been insufficient, which may explain why peritoneal metastasis has become the most common form of recurrent metastasis of GC. Therefore, intraperitoneal therapy was proposed in the third phase (2019–2024) to integrate strategies centered on the removal of free cancer cells from the abdominal cavity into the current treatment paradigm. Hence, the general direction for treatment of GC has integrated novel strategies. In recent years, CRS plus HIPEC have been guided by the concept of regional treatment, with tumor cytology eradication as the standard. By removing visible lesions by CRS and micro-metastatic cancer and free cancer cells with HIPEC, progression of peritoneal metastasis can be effectively controlled to partially realize clinical cure. The results of several clinical trials have shown that CRS with HIPEC can effectively address peritoneal metastasis of GC (37, 43). However, various issues must be addressed in this treatment approach, such as the low quality of evidence of recent clinical studies and the lack of large-sample, high-level evidence-based medical evidence, thereby providing direction for future research.

In conclusion, chemotherapy has been one of the most important areas of research in cancer treatment since the second phase (2011–2019). Advanced technology and increased clinical needs have facilitated continued progress in treatment methods. In this context, HIPEC has gradually received extensive attention. HIPEC, as an emerging therapeutic strategy, is mainly used for treatment of peritoneally disseminated cancers, especially tumors in the digestive system, which have shown good efficacy. With more clinical trials and studies, CRS with HIPEC was confirmed as an effective treatment option for GCPM. This therapeutic strategy not only improves local drug concentrations and reduces systemic toxicities, but also allows the drugs to act more directly on cancer cells, increasing the targeting and effectiveness of the treatment. Henceforth, research and application of CRS with HIPEC should undoubtedly be the focus of future research in cancer treatment to develop more optimized solutions and clinical applications.

## 4 Knowledge framework

**Figure 11:**
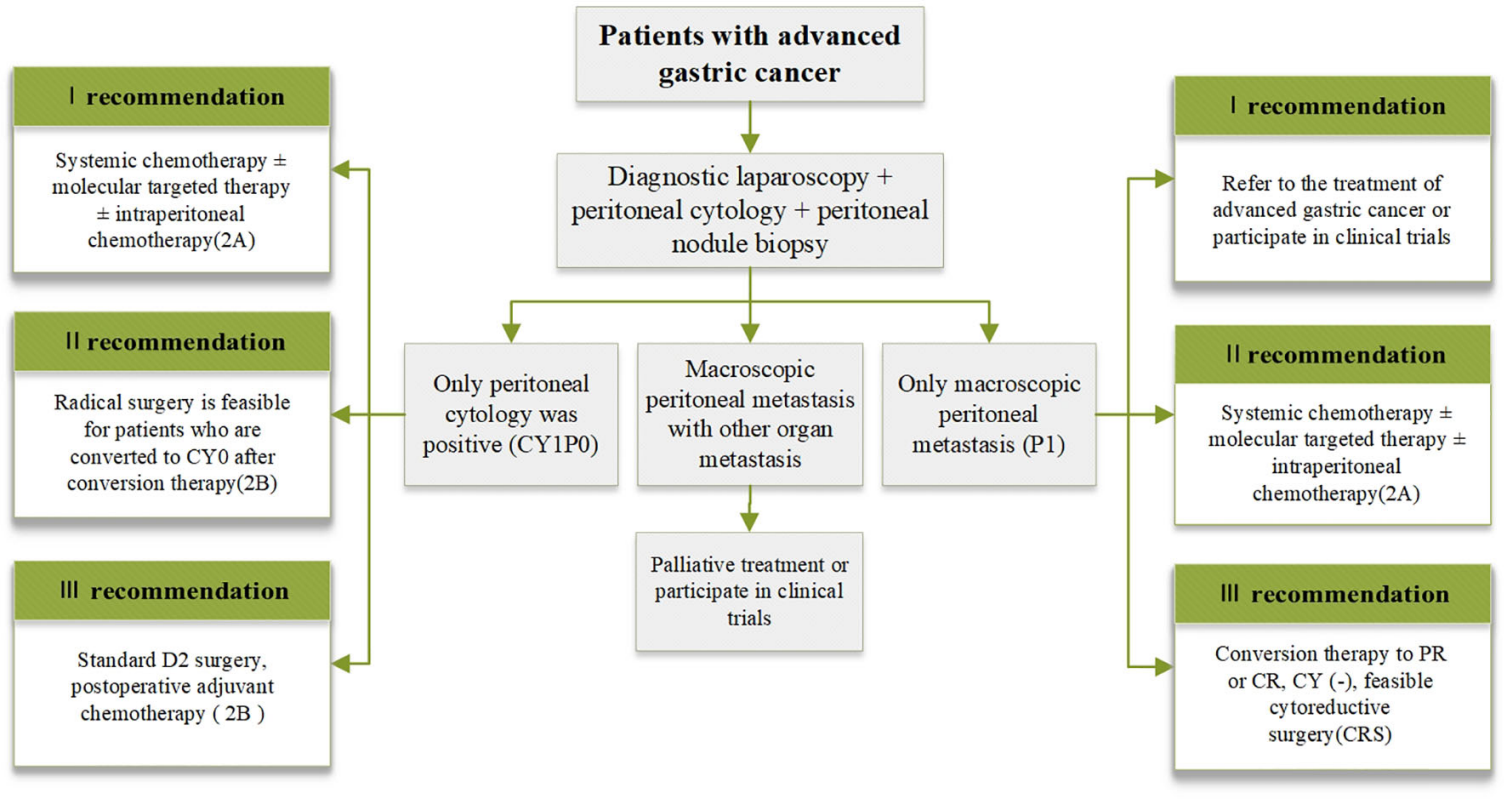
Knowledge framework of the domain.

To facilitate a comprehensive understanding of GCPM studies, it is imperative to offer an overview of recent progress in research. This review attempts to minimize subjectivity by using bibliometric tools and involves a quantity of studies to achieve a holistic outlook on the knowledge base, correlation, status quo, and topic evolution. A knowledge framework was created to visualize the various foci of GCPM research (**Figure 11**).

A knowledge base was quantitatively depicted through the annual trends, as reflected by the total number of publications from 2000 to 2024. Based on the number of publications over the past two decades, there has been a clear upward trend in GCPM research. Henceforth, GCPM is likely to remain a hotspot in the years to come. The top five institutions with the highest number of publications were the National Cancer Center Japan, Nagoya University, Shanghai Jiao Tong University, Aichi Cancer Center, and China Medical University, which are potential candidates for collaboration. Among the top five journals with the highest number of publications, *Annals of Surgical Oncology* (n=58) and *Gastric Cancer* (n=58) should be considered authoritative resources in GCPM research. Further analysis found that the five most productive authors are from either China or Japan.

Knowledge correlation was reflected by cooperation analysis. The results showed that the USA, Japan, France, England, and Germany are closely connected with other countries. China has emerged as a prominent contributor in terms of publications volume, with a notable domestic scholarly collaborations. Kodera, Kitayama, and Yamaguchi are extensively published with strong collaborative ties, thus confirming these authors as leaders in GC research.

The knowledge status quo was indicated by co-citation analysis. The journals *Annals of Surgical Oncology*, *Journal of Clinical Oncology*, *Gastric Cancer*, *EJSO*, and *Carcinoma Research* have the highest co-citation counts and, thus, are valuable resources to assess progress in GC research. The five most co-cited authors were Yonemura, Sugarbaker, Glehen, Ishigami, and Kodera, indicating that these authors have achieved a significant impact in GCPM research.

Knowledge evolution was embodied in keywords. Results of the keywords co-occurrence analysis showed that the incorporated keywords were grouped into five clusters: cluster 1 pertaining to the molecular mechanism of GCPM, cluster 2 focusing on chemotherapy, cluster 3 addressing prognosis, cluster 4 centering on gastric resection, and cluster 5 dealing with intraperitoneal treatment. These five clusters adequately demonstrate the research foci of GCPM research. These results suggest that GCPM research is both a thriving and evolving field, with an undeniable potential for rapid growth in the near future. In the early days of GCPM investigations, the main research direction was gastrectomy. As new techniques and limitations of treatment protocols were exposed, researchers gradually extended studies into chemotherapy, which is expected to be a key research direction in the near future. Meanwhile, treatment targeting the intraperitoneal cavity is gaining increasing attention from the research community. GCPM remains a hot research topic, as evidenced by the findings of this bibliometric analysis, which provides valuable insights for future directions for research endeavors.

## 5 Limitations

However, some limitations must be acknowledged. First, the publications were only sourced from the WOSCC database, suggesting an incomplete search of the literature. Second, this study neglected studies not written in English because of language barriers, which may have resulted in publication bias. Lastly, the selection of disciplinary classifications may have been biased, even though two of the authors independently scrutinized the publications. Notwithstanding these potential limitations, this study established rigorous criteria for screening and analysis of the literature, delineated research hotspots, and forecasted future trends in GCPM research.

## 6 Conclusion

Following a comprehensive bibliometric analysis of the literature on peritoneal metastasis of GC, this study ascertained an increasing focus on GCPM. The results of scholarly output and quality revealed that Japan holds the greatest academic influence, as evidenced by numerous high-quality studies. A combined assessment of publication volume and citation frequency across major journals led to the conclusion that *Annals of Surgical Oncology* has the most significant impact.

Moreover, this study summarized five principal subdomains of GCPM research and synthesized topic evolution via keywords analysis. Notably, research into the mechanisms of GCPM emerged as a hot topic, indicating dynamic development and deepening inquiry within the field. Advancements in prognostic analysis techniques has also garnered increasing recognition among scholars. Such progress fosters mutual reinforcement and the advancement of personalized patient assessment methods (44). The ongoing expansion of surgical indications and the standardization of clinical management practices had significantly prolonged survival of an increasing number of patients (45). Building on this, the application of CRS and HIPEC offer new therapeutic opportunities for patients with GCPM (37). Continuous improvements and updates to CRS/HIPEC protocols are expected to be a focal point of future research. These advancements provide robust support to enhance the survival and quality of life of patients, suggesting that the GCPM research will occupy an even more crucial position in future medical research and practice.
